# Body composition in adults born preterm with very low birth weight

**DOI:** 10.1038/s41390-023-02896-5

**Published:** 2023-11-16

**Authors:** Laura Jussinniemi, Maarit K. Kulmala, Kristina A. D. Aakvik, Silje D. Benum, Anna P. M. Jørgensen, Chandima N. D. Balasuriya, Astrid K. Stunes, Unni Syversen, Marit S. Indredavik, Sture Andersson, Petteri Hovi, Kari Anne I. Evensen, Eero Kajantie

**Affiliations:** 1 Clinical Medicine Research Unit, Oulu University Hospital and University of Oulu, Oulu, Finland; 2 Public Health Unit, Finnish Institute for Health and Welfare, Helsinki, Finland; 3 Helsinki University Eye and Ear Hospital, Helsinki, Finland; 4 Department of Clinical and Molecular Medicine, Norwegian University of Science and Technology, Trondheim, Norway; 5 Department of Neuromedicine and Movement Science, Norwegian University of Science and Technology, Trondheim, Norway; 6 Department of Endocrinology, St. Olavs Hospital, Trondheim University Hospital, Trondheim, Norway; 7 Center for Oral Health Services and Research, Mid-Norway (TkMidt), Trondheim, Norway; 8 Children’s Hospital, Pediatric Research Center, University of Helsinki, Helsinki, Finland; 9 Helsinki University Hospital, Helsinki, Finland; 10 Department of Rehabilitation Science and Health Technology, Oslo Metropolitan University, Oslo, Norway; 11 Children’s Clinic, St. Olavs Hospital, Trondheim University Hospital, Trondheim, Norway

## Abstract

**Background:**

Studies on body composition in preterm very low birth weight (VLBW < 1500 g) survivors are inconsistent and trajectories later in life unknown. We assessed body composition and its change from young to mid-adulthood in VLBW adults.

**Methods:**

We studied 137 VLBW adults and 158 term-born controls from two birth cohorts in Finland and Norway at mean age 36 years. Body composition was assessed by 8-polar bioelectrical impedance. We compared results with dual-energy x-ray absorptiometry measurements at 24 years.

**Results:**

In mid-adulthood, VLBW women and men were shorter than controls. Fat percentage (mean difference in women 1.1%; 95% CI, –1.5% to 3.5%, men 0.8%; –2.0% to 3.6%) and BMI were similar. VLBW women had 2.9 (0.9 to 4.8) kg and VLBW men 5.3 (2.7 to 8.1) kg lower lean body mass than controls, mostly attributable to shorter height. Between young and mid-adulthood, both groups gained fat and lean body mass (*p* for interaction VLBW x age>0.3).

**Conclusion:**

Compared with term-born controls, VLBW adults had similar body fat percentage but lower lean body mass, largely explained by their shorter height. This could contribute to lower insulin sensitivity and muscular fitness previously found in VLBW survivors and predispose to functional limitations with increasing age.

**Impact:**

- In mid-adulthood, individuals born preterm with very low birth weight had similar body fat percentage but lower lean body mass than those born at term. This was largely explained by their shorter height.
- First study to report longitudinal assessments of body size and composition from young to mid-adulthood in very low birth weight adults.
- Lower lean body mass in very low birth weight adults could contribute to lower insulin sensitivity and muscular fitness and lead to earlier functional limitations with increasing age.

## Introduction

Approximately 10% of infants worldwide are born preterm (<37 completed weeks of gestation)^1^ and approximately 1% preterm with very low birth weight (VLBW; <1500 g).^2^ Adults born preterm have increased rates of non-communicable diseases such as type 2 diabetes,^3,4^ coronary heart disease,^5^ hypertension,^4,6–11^ osteopenia or osteoporosis^12,13^ and obstructive airways disease^14^ and higher levels of risk factors of these diseases.^9,10,12,13,15–17^ The mechanisms are unknown, nevertheless alterations in body composition are a good candidate as preterm birth profoundly alters growth of newborn tissues.^6,7^ This may result in altered fat and lean mass, key components of body mass^18^ that are related to the risk of chronic non-communicable diseases^9,13,15,19^ and may also contribute to reduced functional capacity later in life. Follow-up studies report shorter height and smaller head circumference in VLBW adults, indicating impaired skeletal and brain growth.^9,20–22^ Studies also suggest that adults born VLBW or extremely low birth weight (ELBW; <1000 g) have lower lean body mass^9,23,24^ which is partly explained by shorter adult height.^9^ Corresponding associations regarding fat percentage and fat distribution are inconsistent.^9,10,12,20,25–29^

Most studies have only followed VLBW individuals to young adulthood. VLBW infants who first benefited from improved neonatal care are only now approaching middle age, an age when many non-communicable diseases become manifest.^30,31^ In this two-country birth cohort study, we aimed to compare body composition between women and men born with VLBW and term-born controls in mid-adulthood. We assessed trajectories of body composition between young and mid-adulthood and hypothesized that women and men born preterm with VLBW have higher fat percentage and lower lean body mass than their peers born at term and that their fat percentage shows a higher relative increase since the assessment in young adulthood.

## Methods

### Study design

The data were collected by a joint assessment of two longitudinal birth cohorts, the Helsinki Study of Very Low Birth Weight Adults (HeSVA) in Helsinki, Finland, and the NTNU Low Birth Weight in a Lifetime Perspective study (NTNU LBW Life) in Trondheim, Norway. The current mid-adulthood follow-up was carried out in 2019-2021 including a body composition assessment by bioimpedance (BIA) as a part of a comprehensive health assessment.

The original HeSVA cohort comprised 335 VLBW infants, born between January 1978 and December 1985, discharged alive from the neonatal intensive care unit of Helsinki University Central Hospital. For each VLBW infant we selected a singleton term born infant of the same sex and not small for gestational age (SGA), group-matched for sex, age and birth hospital.^9,32^ Both groups have undergone detailed clinical assessments at 22 and 25 years, including body composition assessment at 22 years.^9^ Exclusion criteria for the 22-year body composition assessment were pregnancy, metal in the body and difficulties standing straight.^9^

The NTNU LBW Life cohort comprised 121 VLBW infants born between 1986–1988 who were admitted to the neonatal care unit at St. Olavs Hospital, Trondheim, Norway, and were discharged alive. Non-SGA control participants were born at term to women from the Trondheim region, enrolled before week 20 of their second or third pregnancy in a multicenter study on causes and consequences of intrauterine growth restriction.^33^ SGA participants were excluded from this analysis. The groups have been examined in detail at 1, 5, 14, 20, 23 and 26 years^21,34^ including body composition assessment at 26 years.^12^ Exclusion criteria for the 26-year body composition assessment were pregnancy, congenital syndromes, malformations, and physical disabilities.^12^

Previous young adulthood body composition assessments were completed during 2004-2005 in HeSVA by dual energy x-ray absorptiometry (DXA, Hologic Discovery A, software version 12.3:3, Zaventem, Belgium) at mean age of 22.5 (SD 2.2) and in NTNU LBW Life during 2013-2014 at the mean age of 26.4 (SD 0.6) years by DXA (Hologic Discovery A S/N 83817, Zaventem, Belgium). These assessments have been published separately in either cohort.^9,12,13,35^ We now describe these measurements in the pooled HeSVA-NTNU LBW Life dataset including altogether 421 participants: 116 VLBW women, 91 VLBW men, 124 control women and 90 control men.

### Study participants

**Fig. 1:**
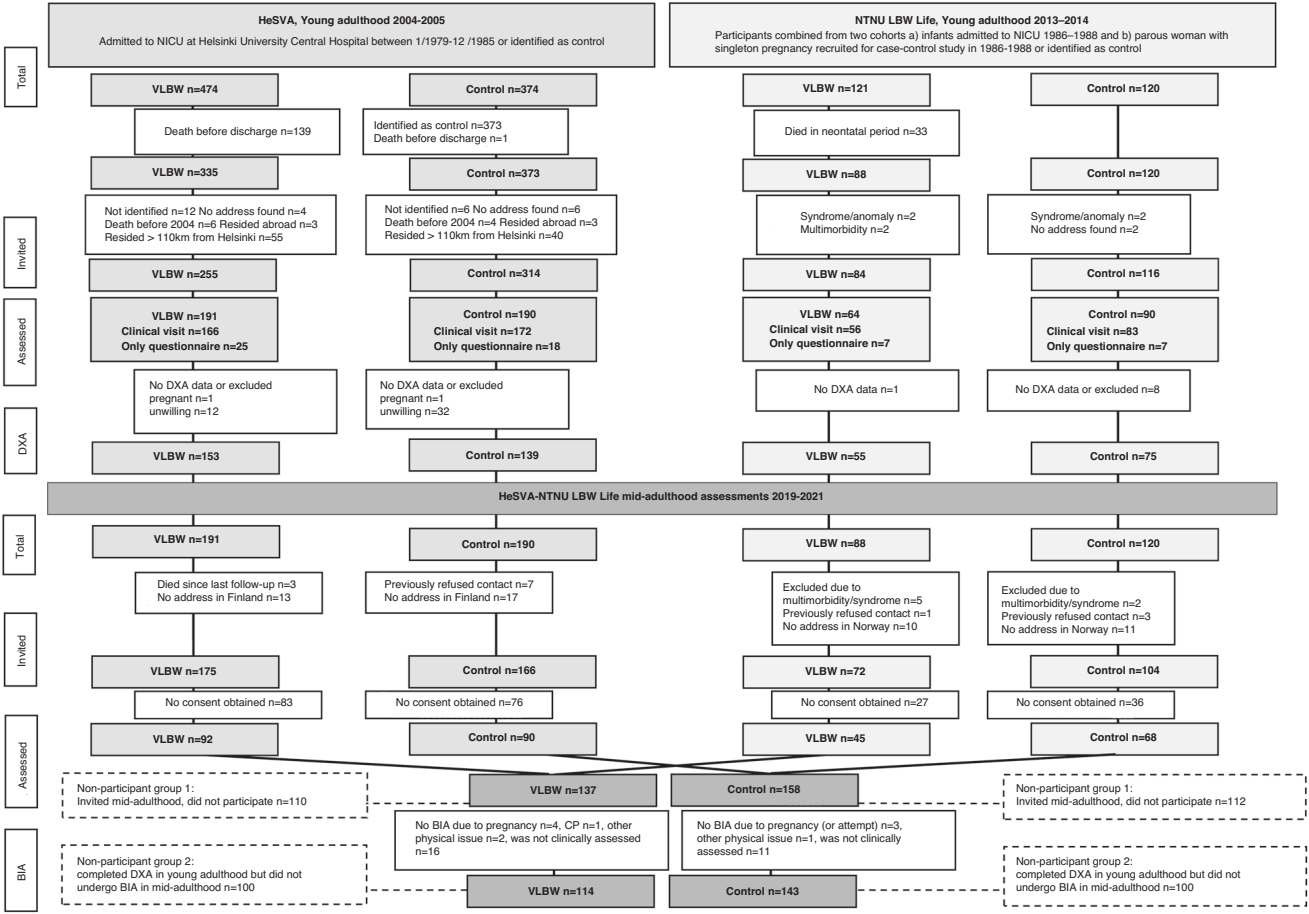
Flow chart from the HeSVA-NTNU LBW Life participants at birth and in young and mid-adulthood assessments.

The flow of study participants from birth through young and mid-adulthood is illustrated in Fig. 1. For the mid-adulthood clinical visits in 2019-2021, we invited 175 VLBW adults from HeSVA and 72 from NTNU LBW Life. Altogether 137 VLBW adults (55.5%; 79 women, 58 men) attended the study, and 114 (46.2%; 65 women, 49 men) completed bioimpedance assessment. For the control group, we invited 166 from HeSVA and 104 form NTNU LBW Life. Altogether 158 control participants attented (58.5%; 93 women, 65 men) and 143 (53.0%; 81 women, 62 men) completed the bioimpedance assessment (Fig. 1). Exclusion criteria for the bioimpedance assessment were pregnancy, metal in the body or difficulties standing straight.

All participants gave their written informed consent. All involved in the data collection were blinded for the participants’ group, birth weight and other relevant neonatal characteristics. The study protocols were approved by the ethics committee at the Helsinki and Uusimaa Hospital District (HUS/1157) and by the Regional Committee for Medical and Health Research Ethics in Central-Norway (23879). The protocol is registered as ISRCTN77533991.

### Measurements

The mid-adulthood anthropometric measurements included height, waist, hip and head circumference based on the European Health Examination Survey field work manual.^36^ Body weight and composition was examined by 8-polar bioelectrical impedance analysis (Seca® mBCA 515, Hamburg, Germany). Bioelectrical analysis was not performed if the participant was pregnant, had metal in the body or refused. To ensure similar measurements at the two research sites, audits were carried out before and during the data collection.

Height was measured using fixed stadiometer with the participant standing evenly on both feet in a normal standing position. The mean of the three measurements was used in analysis.^36^

Head circumference was measured with non-elastic measuring tape in the widest circumference above the ears and brows. The maximum of the three measurements was used in analysis.^36^

Waist circumference was measured midway between the lower rib and the iliac crest from bare skin with non-elastic measurement tape. Measurement was not performed if the participant was in a wheelchair, could not stand straight or was pregnant (>20 weeks). The mean of the two measurements was used in analysis.^36^

Hip circumference was measured with non-elastic measurement tape over the buttock at the maximal circumference. The mean of the two measurements was used in analysis.^36^

The anthropometric measurements in young adulthood were carried out largely with similar methods and body composition assessment was performed by DXA.^12,13^

### Statistical analyses and power calculation

We analyzed data with IBM SPSS Statistics (Chicago, Illinois), Version 28.0. We assessed normality and distributions by evaluating histograms and Q-Q Plot residuals. We used linear regression models to analyze group differences between VLBW adults and controls at each time point and mixed models to analyze changes in group differences between young and mid adulthood. We performed separate analyses by sex. All analyses were adjusted for cohort and age. For fat free mass, we ran a separate model adjusting also for height. We used bias corrected and accelerated (BCa) bootstrapping method with B = 2000 bootstrap samples in all the analysis except for the mixed model analysis of the VLBW x age interaction. A priori power calculation was based on a total population of 170 VLBW participants and 200 controls. With a statistical power of 80% and alpha level of 0.05, the detectable difference between the groups was 0.29 SD score and with 90% power and alpha of 0.01 the detectable difference was 0.40 SD. Before data analysis, with the actual number of 137 VLBW and 158 control participants, the corresponding numbers were 0.33 and 0.45 SD.

## Results

### Background characteristics of the mid-adulthood clinical visit

Main background and maternal characteristics are described in Table 1. The VLBW participants were born at mean 29.4 (SD 2.4) weeks of gestation with a mean birth weight of 1169 (SD 214) grams, while control participants were born at mean 40.0 (SD 1.2) weeks of gestation weighing 3660 (SD 479) grams. Adults born with VLBW had lower educational attainment than controls (*p* = 0.02).

**Table 1:** Background characteristics of participants born with VLBW or at term in mid-adulthood.

	VLBW	Term control	VLBW vs. control *P* value
Number of participants*	114	143	
HeSVA	79	85	
NTNU	35	58	
**Maternal background characteristics**	**Mean (SD) or** ***n*** **(%)**	**Mean (SD) or** ***n*** **(%)**	
Height (cm)	164.9 (6.3)	165.8 (5.8)	0.24
BMI (kg/m^2^)	22.9 (3.5)	22.9 (3.2)	0.90
Smoking during pregnancy**	13 (22.8%)	11 (16.7%)	0.39
Parental educational attainment			0.051
basic or less	21 (19.1%)	13 (9.8%)	
upper secondary	20 (18.2%)	30 (22.7%)	
lower-level tertiary	38 (34.5%)	36 (27.3%)	
upper-level tertiary	31 (28.2%)	53 (40.2%)	
**Study participant background characteristics**			
Sex, women	65 (57.0%)	81 (56.6%)	0.95
Gestational age (weeks)	29.4 (2.4)	40.0 (1.2)	<0.001
Birth weight (g)	1169 (214)	3660 (479)	<0.001
Birth weight SD score Finnish reference	–1.2 (1.7)	0.2 (1.0)	<0.001
Birth wight SD score Norwegian reference	–1.0 (1.2)	0.1 (1.0)	<0.001
C-section as a delivery mode	75 (66.4%)	13 (15.3%)	<0.001
Cerebral palsy	2 (1.8%)	–	0.21
Ventilator treatment (days)	7.8 (13.6)	–	
Supplemental oxygen (days)	21.1 (41.5)	–	
Age at discharge from hospital (days)	69.9 (43.4)	–	
Bronchopulmonary dysplasia			
defined as supplementary oxygen at more than 28 days	29 (27.1%)	–	
defined as supplementary oxygen at more than 36 weeks	8 (7.5%)	1	
diagnosed by clinician	19 (23.5%)	–	
**Study participant current characteristics**			
Age (years)	36.3 (3.2)	35.8 (3.3)	0.26
Educational attainment			0.02
lower (ISCED levels 1-2)	4 (3.5%)	2 (1.4%)	
Intermediate (ISCED levels 3-5)	54 (47.4%)	47 (32.9%)	
lower tertiary or higher (ISCED levels 6-8)	56 (49.1%)	94 (65.7%)	

*Among the participants who completed the bioimpedance assessment (*n* = 257).

**Data only available from HeSVA.

*HeSVA* the Helsinki Study of Very Low Birth Weight Adults, *ISCED* international standard classification of education, *NTNU* NTNU Low Birth Weight in a Lifetime Perspective Study, *SD* standard deviation, *VLBW* very low birth weight.

### Body composition in mid-adulthood

The anthropometry and body composition results are described in Table 2. Compared with controls, VLBW women were 4.1 (95% CI: 1.5 to 6.4) cm and men 6.1 (95% CI: 3.6 to 8.5) cm shorter, and they had smaller head circumference. There were no group differences in fat percentage, fat mass and BMI. Among women, 12 (18.5%) VLBW, and 25 (30.9%) control participants were overweight, and 18 (27.7%) VLBW and 15 (18.5%) control participants were obese. Among men, overweight was observed in 18 (36.7%) VLBW, and 24 (38.7%) control participants, and 9 (18.4%) VLBW and 6 (9.7%) control participants were obese. Differences in overweight and obesity were not statistically significant. Adults born with VLBW had lower lean body mass. However, lean body mass adjusted for height showed no difference between the groups.

**Table 2:** Comparison of development of body size and composition from young adulthood to mid-adulthood between the VLBW and control participants.

	VLBW women	Control women	Mean difference* (95% CI)		Age Interaction** *P*-value	VLBW men	Control men	Mean difference (95% CI)	Age interaction *P* value
Young adulthood n	115	124				91	90		
Current study n	65	81				49	62		
	**Mean (SD) or** ***n*** **(%)**	**Mean (SD) or** ***n*** **(%)**				**Mean (SD) or** ***n*** **(%)**	**Mean (SD) or** ***n*** **(%)**		
Age (years)									
young adulthood	23.3 (2.4)	23.9 (2.6)				23.5 (2.7)	23.9 (2.6)		
mid-adulthood	36.1 (3.2)	36.0 (3.4)				36.5 (3.3)	35.6 (3.1)		
Height (cm)									
young adulthood	162.7 (7.7)	167.2 (6.5)	–4.3 (–6.1 to –2.5)			175.9 (7.3)	181.9 (6.2)	–5.7 (–7.6 to –3.6)	
mid-adulthood	163.3 (8.0)	167.5 (6.2)	-4.1 (-6.5 to –1.6)		0.06	175.7 (6.7)	182.1 (6.4)	–6.1 (–8.4 to –3.7)	0.61
Head circumference (cm)									
mid-adulthood	54.8 (1.6)	56.1 (1.3)	–1.2 (–1.7 to –0.8)			57.1 (1.9)	58.3 (1.1)	–1.1 (–1.7 to –0.6)	
Weight (kg)									
young adulthood	60.7 (13.2)	66.0 (12.0)	-4.8 (–7.9 to –1.8)			71.8 (14.8)	81.2 (12.4)	–8.7 (–12.7 to –5.1)	
mid-adulthood	69.9 (18.1)	72.2 (14.8)	–2.6 (–8.3 to 3.2)		0.57	78.9 (15.3)	84.4 (12.6)	–5.4 (–10.8 to –0.3)	0.25
Mean difference mid vs young adulthood, (95% CI)***	8.2 (5.9 to 10.5)	6.9 (4.6 to 9.2)				7.2 (4.6 to 9.8)	4.4 (2.1 to 6.8)		
BMI									
young adulthood	22.7 (4.6)	23.4 (4.1)	–0.5 (–1.6 to 0.6)			22.8 (4.0)	24.2 (3.5)	–1.2 (–2.2 to –0.1)	
mid-adulthood	26.2 (6.6)	25.7 (5.0)	0.3 (–1.5 to 2.2)		0.33	25.6 (4.8)	25.4 (3.4)	0.1 (–1.5 to 1.7)	0.20
Mean difference mid vs young adulthood, (95% CI)***	3.4 (2.5 to 4.2)	2.6 (1.8 to 3.4)				2.7 (1.8 to 3.5)	1.7 (1.1 to 2.4)		
Fat mass (kg)									
young adulthood	18.8 (8.0)	19.7 (7.1)	–.7 (–2.7 to 1.2)			13.5 (6.8)	15.6 (6.8)	–1.8 (–3.7 to 0.0)	
mid-adulthood	26.1 (12.3)	25.6 (10.4)	0.3 (–3.6 to 4.3)		0.68	20.0 (10.9)	19.6 (7.9)	–0.1 (–3.6 to 3.4)	0.33
Mean difference mid vs young adulthood, (95% CI)***	7.0 (5.5 to 8.6)	6.3 (4.7 to 7.9)				6.7 (4.8 to 8.6)	4.8 (3.0 to 6.6)		
Fat percentage (%)									
young adulthood	30.0 (6.5)	29.3 (6.1)	0.7 (–1.0 to 2.3)			18.1(6.0)	18.6 (5.7)	–0.5 (–2.3 to 1.4)	
mid-adulthood	35.6 (8.4)	34.2 (7.4)	1.1 (–1.5 to 3.5)		0.73	24.1 (9.1)	22.7 (6.3)	0.8 (–2.0 to 3.6)	0.31
Mean difference mid vs young adulthood, (95% CI)***	6.0 (4.8 to 7.1)	5.5 (4.4 to 6.6)				6.3 (4.5 to 8.0)	4.6 (3.2 to 6.0)		
Lean body mass (kg)									
young adulthood	40.4 (6.4)	44.8 (6.3)	–3.8 (–5.3 to –2.4)			56.6 (10.3)	64.0 (8.3)	–6.7 (–9.2 to –4.2)	
mid-adulthood	43.8 (6.7)	46.7 (5.7)	–2.9 (–4.8 to –0.9)		0.58	58.8 (7.6)	64.7 (7.3)	–5.3 (–8.1 to –2.7)	0.41
Mean difference mid vs young adulthood, (95% CI)***	2.6 (1.6 to 3.6)	1.8 (0.8 to 2.8)				2.1 (0.7 to 3.5)	1.0 (–0.1 to 2.1)		
Lean body mass (kg) adjusted for height									
young adulthood	41.8 (4.8)	43.4 (4.9)	–1.9 (–3.2 to –0.7)			59.4 (6.9)	61.3 (6.9)	–2.3 (–4.6 to 0.3)	
mid-adulthood	44.9 (5.9)	45.8 (4.6)	–1.0 (–2.7 to 0.9)		0.39	61.5 (5.8)	62.7 (6.0)	–1.5 (–4.0 to 1.0)	0.29
Mean difference mid vs young adulthood, (95% CI)***	2.6 (1.7 to 3.6)	1.7 (0.7 to 2.7)				2.2 (0.9 to 3.6)	0.9 (–0.2 to 1.9)		
Waist circumference (cm)									
young adulthood	78.8 (11.0)	79.9 (9.6)	–0.7 (–3.3 to 1.8)			84.3 (10.4)	88.3 (9.3)	–3.6 (–6.5 to –0.7)	
mid-adulthood	84.5 (14.7)	82.9 (10.7)	1.3 (–2.6 to 5.6)		0.33	91.3 (12.8)	90.1 (9.7)	0.7 (–3.4 to 5.0)	**0.03**
Mean difference mid vs young adulthood, (95% CI)***	5.4 (3.0 to 7.7)	3.5 (1.6 to 5.5)				7.0 (4.4 to 9.7)	3.0 (1.0 to 4.9)		
Hip circumference (cm)									
young adulthood	83.5 (15.4)	87.1 (14.6)	–1.4 (–4.1 to 1.2)			88.3 (13.2)	94.3 (11.7)	–4.3 (–6.9 to –1.7)	
mid-adulthood	101.5 (12.9)	101.6 (12.9)	–0.2 (–3.9 to 3.4)		0.44	98.0 (8.2)	100.7 (7.0)	–2.4 (–5.3 to 0.3)	0.60
Mean difference mid vs young adulthood, (95% CI)***	16.3 (13.2 to 19.4)	13.5 (10.5 to 16.4)				9.9 (6.9 to 12.9)	6.9 (4.7 to 9.1)		

*Mean difference is calculated with linear regression, adjusted for cohort and age.

**Interaction between VLBW birth and visit (young or mid-adulthood) is assessed by a mixed model, adjusted for cohort and age.

***Mean difference between young and mid-adulthood visit is assessed by mixed model, adjusted for cohort.

*CI* confidence interval, SD standard deviation, *VLBW* very low birth weight

### Body composition in young adulthood and its development to mid-adulthood

**Fig. 2:**
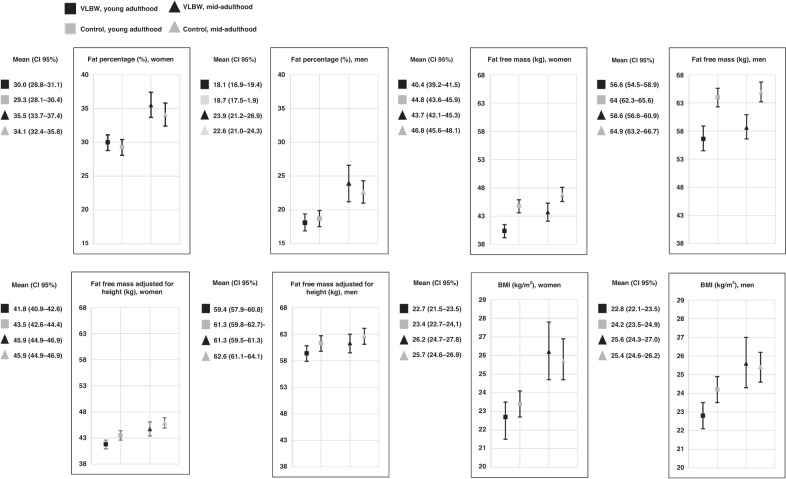
Main body composition outcomes (mean, 95% CI) in adults born preterm with VLBW compared to term-born controls in young and mid-adulthood.

The young adulthood anthropometric and body composition outcomes are described in Table 2 and Fig. 2. VLBW men and women were shorter and had smaller heads than controls. VLBW men had 1.2 (95% CI: 0.1 to 2.2) kg/m^2^ lower BMI than control men, whereas there was no difference in BMI among women (Table 2). In addition, VLBW men had a smaller hip and waist circumference. Both men and women in the VLBW group had lower lean body mass, but after adjustment for height, the result remained statistically significant only for VLBW women (Fig. 2, Table 2).

Among men, we found a significant VLBW x age (young or mid-adulthood) interaction in waist circumference (*p* = 0.03). In young adulthood, VLBW men had smaller waist circumference (mean difference 3.6 cm, 95% CI: 0.7 to 6.5) compared with control men. Waist circumference increased on average by 7.0 cm in VLBW men and 1.8 cm in control men, such that the difference was no longer seen in mid-adulthood (mean difference 0.7 cm; 95% CI: -3.4 to 5.0; Table 2). Otherwise, we found no significant VLBW x age interactions (Table 2). Participants in both groups gained weight between young and mid-adulthood but differences in their body composition remained largely similar between the two ages.

### Non-participants

We conducted two sets of non-participant analyses. First, the participants who attended the mid-adulthood clinical visit were compared with those who were invited but did not attend (Supplementary table 1). In this non-participant analysis, maternal BMI was lower among non-participating controls (mean 21.6, SD 2.7) than participating controls (mean 22.8, SD 3.2, *p* = 0.004).

Second, we carried out a non-participant analysis comparing participants who attended both the young and mid-adulthood clinical visit with those who completed DXA assessment in young adulthood but did not attend BIA assessment in the mid-adulthood (Supplementary table 2). Control men who attended DXA assessment in young adulthood but did not attend BIA in mid-adulthood had lower BMI and lean body mass adjusted for height in young adulthood compared with control men who participated at both time points. There were no other differences between non-participants and participants.

## Discussion

### Main findings

In mid-adulthood women and men born preterm with very low birth weight were shorter and had smaller head circumference than their peers born at term. They also had lower lean body mass which was largely explained by their shorter height. While both the VLBW and term-born participants gained weight between young and mid-adulthood, differences in body composition between the groups remained largely similar or, if anything, became smaller.

### Study strengths and limitations

We conducted the mid-adulthood measurements in two birth cohorts with harmonized methods, which increased power and added precision. Assessors were blinded to birth status. We carried out audits before and during data collection to ensure similar measurements.

As to limitations, we used two different measurement techniques to measure body composition. In young adulthood body composition was assessed by DXA which is based on the three-compartment model of body composition comprising fat, lean tissue mass and bone mineral In mid-adulthood we used 8-polar bioelectrical impedance analysis, based on two-compartment-model comprising fat and lean mass without distinguishing between bone mineral and other lean mass.^37^ The absolute differences in body composition between young and mid-adulthood should thus be treated with caution. However, our conclusions are mainly based on differences between VLBW and control groups, where the same measurement technique was used at the same time point. Many studies have investigated and compared these two body composition measurement techniques;^37–42^ DXA is accurate^43^ and is considered as a reference method in clinical research, but it is expensive and requires special radiological equipment.^44^ Bioelectrical impedance analysis is a non-invasive, simple, low-cost, safe technique^45^ and is a widely used method for epidemiological and clinical purposes^40^ and particularly useful in comparing differences between groups,^41^ although it does not distinguish bone from other lean tissues.

### Consistency with previous research

Our study is the first to report longitudinal assessments of body size and composition from young to mid-adulthood. Body composition differences between VLBW adults and controls remained largely similar between these ages. Previous findings that VLBW young adults have lower lean body mass, which was largely attributable to shorter height^35,46^ was also observed in mid-adulthood. No change in fat percentage or BMI seemed to emerge between young and mid-adulthood. The only statistically significant interaction between group and age was for men’s waist circumference, where waist circumference increased more rapidly among VLBW than control men, such that no difference was any longer seen in mid-adulthood.

Findings on fat percentage and BMI among adults born preterm vary between studies. Contrary to our findings, some studies have reported higher fat percentages among VLBW adults.^10,46–48^ A systematic review by Markopoulou et al. included nine studies estimating fat percentage measured by BIA, DXA or whole body magnetic resonance imaging (MRI) in preterm born adults compared with term-born controls. Results showed higher fat percentage both in random effect (mean difference 1.5 percentage points, *p* = 0.03) and fixed effect (mean difference 1.2 percentage points, *p* = 0.009) models.^47^ This difference falls within our confidence intervals and would not have been observed in the present study. However, there was moderate heterogeneity, and the difference was largely attributable to three of the nine studies. Two of the studies showing higher fat percentage included adults born at any degree of prematurity, most of whom are were late preterm, 34-36 completed weeks.^28,49^ Together with another study^10^ not included in the meta-analysis suggests that increased fat percentage is a feature of adults born late preterm but not earlier preterm. However, one of the studies included in the meta-analysis showed higher fat percentage in extremely low birth weight adults.^48^ This is partly consistent with another study using air displacement plethystomography showing that men but not women born at 33 weeks or less had 13% higher fat percentage, whereas men or women born late preterm showed no difference compared with controls.^46^ Reasons for these discrepancies are not known.

Our finding of no difference in fat percentage does not exclude differences in fat distribution. Putting on weight causes adipocytes to enlarge and increases not only subcutaneous fat but also fat deposits in other vulnerable areas of the body. This ectopic fat is deposited in the intra-abdominal visceral fat depot, in muscle, in the liver and in the beta cells. On a population level, waist circumference is a commonly used indicator of abdominal fat and is a strong predictor of cardiovascular mortality.^50^ Studies on young adults born preterm in our source cohorts HeSVA and NTNU^9,21^ have reported lower waist circumference, which has not been confirmed in other studies.^48,51^ Our findings suggest that at least among men any difference in waist circumference in young adult age seems to level off by mid-adulthood. Visceral fat can be more accurately measured by MRI. One MRI study reported that 23 adults born at <33 weeks have higher visceral fat and hepatocellular fat, than 29 term-born controls,^51^ but all analyses were adjusted for BMI making the significance of the finding unclear. Another MRI study found no difference in visceral fat area between 29 extremely low birth weight adults and 13 controls. A study of 78 VLBW adults and 72 sibling-controls reported no difference in visceral fat volume or hepatocellular or muscle fat.^52^

Our findings related to lean body mass are largely in line with previous research. As others,^9,13,23,48^ we found lower lean body mass among VLBW adults. After height adjustment the results did not remain statistically significant.

### Clinical implications

Lean body mass tracks across life course^24^ and usually starts to decline 3-8% per decade after the age of 30 years.^53^ A major component of lean body mass is muscle, and lower amount of muscle could contribute to reduced muscular fitness^54^ and insulin sensitivity^19^ previously shown in adults born preterm. Lower lean mass and higher fat percentage have also been associated with lower bone mineral density lower,^55^ although it is uncertain whether body composition predicts bone fractures over and above the risk associated with other clinical risk factors.^56^ As VLBW adults have lower lean body mass in their young and mid-adulthood, this normal decline in lean body mass could be expected to cause reduced functional capacity and predispose to various non-communicable diseases^3,5,14^ earlier than among adults born at term.

However, in our study the difference in lean body mass remained similar in young and mid-adulthood, and we found no difference in the speed of decline in lean body mass between VLBW adults and controls.

While we found no difference in fat percentage, other risk factors, such as higher blood pressure^21,22^ and reduced insulin sensitivity^9,19,28^ in adults born preterm may underlie the increased risk non-communicalbe diseases.^9,13,15,19^

In conclusion, adults born with very low birth weight entering middle age were shorter and had smaller head circumference than adults born at term. In addition, they had lower lean body mass largely attributable to their shorter height. There was no difference in body fat percentage or BMI. While the participants gained weight between young and mid-adulthood, differences in body composition remained largely similar. Lower lean body mass could contribute to lower insulin sensitivity and muscular fitness and lead to earlier functional limitations with increasing age.

### Supplementary information

Supplementary Table 1

Supplementary Table 2

## Data Availability

The datasets generated and/or analyzed during the current study include sensitive health data and cannot be made publicly available. Aggregated data is available from the corresponding author on reasonable request.
